# Prurigo nodularis in vitiligo patches: An unusual observation

**DOI:** 10.1016/j.jdcr.2020.06.039

**Published:** 2020-07-01

**Authors:** Karim Saleh

**Affiliations:** Division of Dermatology and Venereology, Department of Clinical Sciences, Lund University, Skåne University Hospital, Lund, Sweden

**Keywords:** itch, prurigo nodularis, pruritus, vitiligo, PN, prurigo nodularis

## Introduction

Prurigo nodularis (PN), first described in 1909 by Hyde and Montgomery,^1^ is a chronic skin disorder presenting with symmetrically distributed, multiple, firm, intensely pruritic nodules. Its etiology remains unknown, but a frequent association seen is atopic dermatitis.^2^ Other less common associations are pregnancy, internal diseases, malabsorption, or malignancies.^3^ We report a case of PN occurring in vitiligo patches in an 82-year-old patient.

## Case report

An 82-year-old man with a history of alopecia totalis, vitiligo, and PN has been a patient at our department for the last 10 years. He has no other medical conditions or psychiatric history. Alopecia totalis developed in our patient when he was approximately 40 years of age.

At an unspecified interval after this development, generalized vitiligo affecting the face, abdomen, and extremities developed. Several years after the onset of vitiligo, pruritus developed. It was then that the patient presented to our department. PN diagnosis was confirmed by histopathology. Routine laboratory workup and radiologic examinations including a chest radiograph and abdominal ultrasound scan found no secondary cause of his pruritus.

The patient had severe pruritus. Throughout the course of the condition, he had received various treatments for PN, initially topical steroids, then tacrolimus and phototherapy, and finally oral methotrexate with varying doses. He was not interested in treating his vitiligo or alopecia. He is currently being treated with methotrexate, 15 mg weekly, with an unsatisfactory effect on his pruritus. Currently, he is not using any topical therapy, emollients, or sun protection.

**Fig 1:**
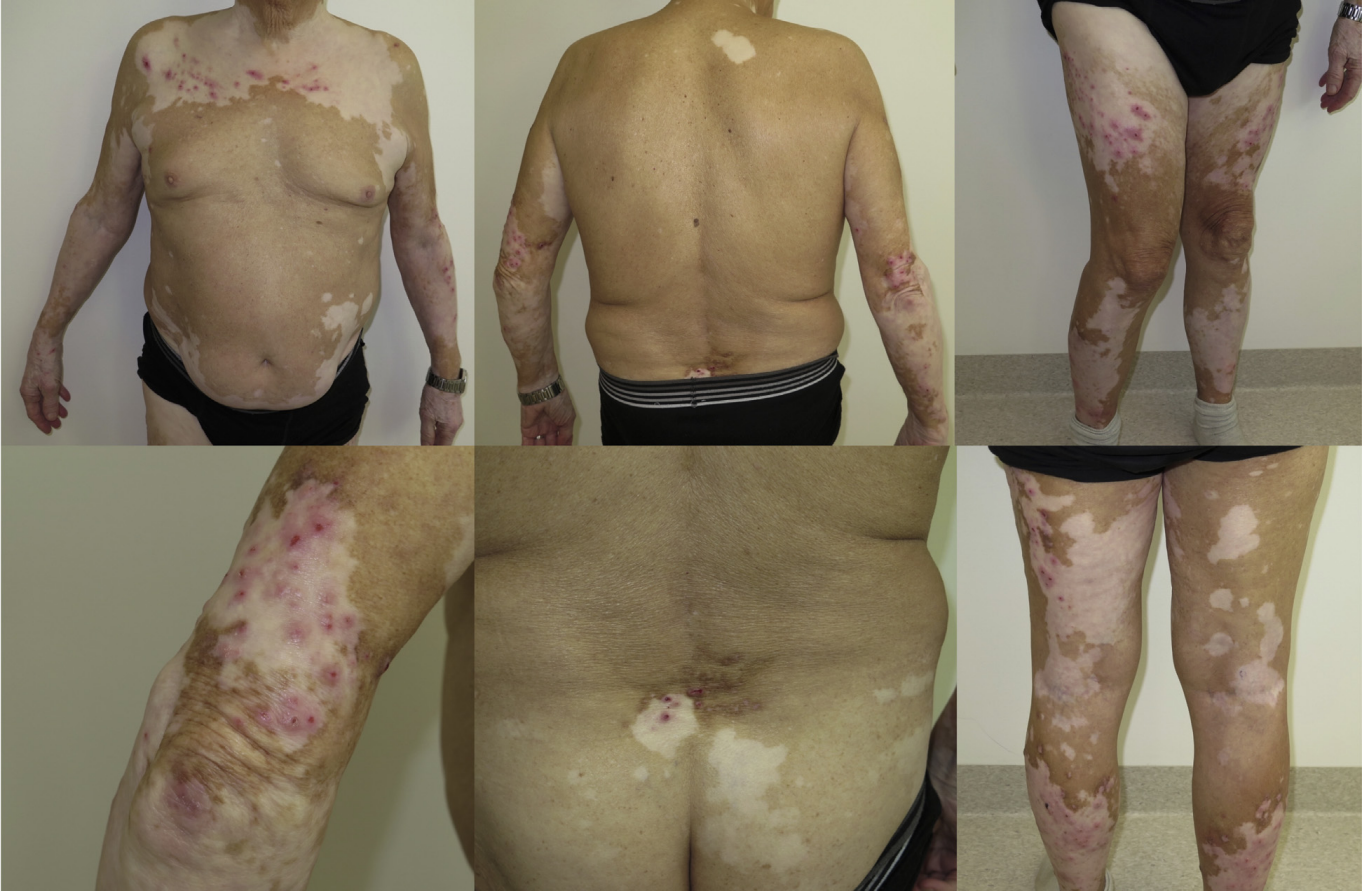
Prurigo nodules occurring in vitiligo patches. Nonvitiligo skin had no prurigo nodules.

It was not until the most recent clinic visit that it was noticed that all PN nodules occurred in vitiliginous patches (Fig 1). His vitiligo has followed a stable course throughout the years. He has no facial or genital PN but does have vitiligo at these sites. He has no pruritus or PN on nonvitiliginous skin.

## Discussion

To the best of our knowledge, this is the first report of PN occurring in vitiligo. Vitiligo is a depigmentation disorder of the skin, hair, or mucosa showing white macules or patches caused by the destruction of melanocytes.^4^ The pathogenesis of vitiligo is still not fully understood. A recent hypothesis focuses on a neurogenic mechanism with the release of melanocyte-toxic neuropeptides of cutaneous peripheral nerve endings.^5^ This finding might explain the occurrence of pruritus in vitiligo patients. Pruritus in vitiligo was first reported in 1958.^6^ Published data on the prevalence and characteristics of itch in vitiligo are very limited. In a study of 245 patients with generalized vitiligo, 20% of patients had pruritus.^7^ In another study of 402 patients with vitiligo, 28% of patients experienced mild pruritus, 43% moderate pruritus, and 28% severe pruritus. Pruritus was one of the clinical signs of disease activity when assessing vitiligo in a recent metanalysis.^8^ Because it appears pruritus is fairly common in vitiligo and can sometimes be severe, not to mention the existing similar hypotheses of etiologies mentioned above, we postulate that prurigo nodules in our patient were secondary to his vitiligo, an observation that has never been reported previously. Clearly, further studies are needed to examine and assess this association.

## References

[1] Hyde J.N. (1888). A Practical Treatise on Diseases of the Skin: For the Use of Students and Practitioners.

[2] Lee M.R., Shumack S. (2005). Prurigo nodularis: a review. Australas J Dermatol.

[3] Wallengren J. (2004). Prurigo: diagnosis and management. Am J Clin Dermatolo.

[4] Boissy R.E., Manga P. (2004). On the etiology of contact/occupational vitiligo. Pigment Cell Res.

[5] Zeidler C., Pereira M.P., Huet F. (2019). Pruritus in autoimmune and inflammatory dermatoses. Front Immunol.

[6] Levai M. (1958). The relationship of pruritus and local skin conditions to the development of vitiligo. AMA Arch Dermatol.

[7] Linthorst Homan M.W., Spuls P.I., de Korte J. (2009). The burden of vitiligo: patient characteristics associated with quality of life. J Am Acad Dermatol.

[8] van Geel N., Grine L., De Wispelaere P. (2019). Clinical visible signs of disease activity in vitiligo: a systematic review and meta-analysis. J Eur Acad Dermatol Venerol.

